# Antibacterial activities of chemical constituents from the aerial parts of *Hedyotis pilulifera*

**DOI:** 10.1080/13880209.2017.1279673

**Published:** 2017-01-19

**Authors:** Hoai Thi Nguyen, Duc Viet Ho, Hung Quoc Vo, Anh Tuan Le, Hien Minh Nguyen, Takeshi Kodama, Takuya Ito, Hiroyuki Morita, Ain Raal

**Affiliations:** a Faculty of Pharmacy, Hue University of Medicine and Pharmacy, Hue University, Hue City, Vietnam;; b Quang Tri Center of Science and Technology, Mientrung Institute for Scientific Research, Quang Tri, Vietnam;; c Institute of Natural Medicine, University of Toyama, Toyama, Japan;; d Institute of Pharmacy, University of Tartu, Tartu, Estonia

**Keywords:** Oleanolic acid, rotungenic acid, rotundic acid, *Staphylococcus aureus*; *Bacillus subtilis*; *Mycobacterium smegmatis*

## Abstract

**Context:***Hedyotis pilulifera* (Pit.) T.N. Ninh (Rubiaceae) has been used in Vietnamese ethnomedicine; the methanol extract exhibited antibacterial activity in our preliminary screening.

**Objectives:** In this study, compounds from *H. pilulifera* were isolated and their antibacterial activity *in vitro* was evaluated.

**Materials and methods:** The aerial parts of *H. pilulifera* (1.4 kg) were extracted with MeOH, suspended in water and ethyl acetate extract was chromatographed on a silica gel column. The structures of isolated compounds were elucidated by the combination analyses of spectroscopy including 1D-, 2D-NMR, HRMS and in comparison with the reported NMR data in the literature. All isolated compounds were evaluated for inhibitory effect using the microdilution method toward *Staphylococcus aureus, Bacillus subtilis* and *Mycobacterium smegmatis,* and MIC values were determined.

**Results:** Twenty compounds were isolated, including five triterpenoids, two steroids, two aromatic compounds, three fatty acids, one quinone derivative, one lignan glycoside, one ceramide and five glycolipids. Among these, oleanolic acid showed significant antibacterial activity against *M. smegmatis* with the MIC value of 2.5 μg/mL. Remarkably, rotungenic acid showed strong activity against *S. aureus, B. subtilis, M. smegmatis* with MIC values of 2.5, 2.5 and 1.25 μg/mL, respectively. Rotundic acid exhibited significant antibacterial activity against *B. subtilis* with the MIC value of 5 μg/mL. To the best of our knowledge, the antibacterial activity of rotungenic acid, stigmast-4-ene-3,6-dione and (2*S*,3*S*,4*R*,2′*R*)-2-(2′-hydroxytetracosanoylamino) octadecane-1,3,4-triol was reported for the first time.

**Conclusions:** Oleanolic acid, rotungenic acid, and rotundic acid were considered to be useful for developing new antimicrobial therapeutic agents for human.

## Introduction

*Hedyotis pilulifera* (Pit.) T.N. Ninh (Rubiaceae), an herbal plant, is known to be distributed in Laos and Vietnam (Ho 2003; Newman et al. 2007). It has been used as the remedy for abdominal pain and osteoarthritis (Bossière et al. 2006). The valuable experience of ethnomedicine should not to be underestimated: for example, according to our studies, the needles of *Pinus sylvestris* L. (Pinaceae) showed cytotoxic effect against breast cancer cells (Hoai et al. 2015), they have been used against cancer in Estonian ethnomedicine (Sak et al. 2014).

In our previous report, one new iridoid aglycone, 10-*O*-acetylborreriagenin, and five known iridoidial glycosides were isolated from the aerial parts of *H. pilulifera* (Hoai et al. 2016). Generally, the chemical composition of genus *Hedyotis,* including *H. pilulifera,* has been investigated just to a small extent. In our preliminary unpublished data, the methanol extract of aerial parts of *H. pilulifera* showed significant antimicrobial activity. Therefore, the current study was conducted to isolate those compounds and to evaluate their antibacterial activity *in vitro*.

## Materials and methods

### General

Melting points were determined on a Yanaco Micro MP apparatus. Optical rotations were measured on a JASCO P-2100 polarimeter (Hachioji, Tokyo, Japan). Infrared spectra were recorded on JASCO FT/IR-460 Plus spectrometer. 1D and 2D NMR were carried out using Bruker Avance 500 spectrometer (Bruker, Mass., Billerica, MA) with tetramethylsilane as an internal standard. ESI-MS data including high-resolution mass spectrum were measured on Shimadzu LCMS-IT-TOF spectrometer (Kyoto, Japan) HREIMS was recorded on a JEOL MStation JMS-700 spectrometer (JEOL Ltd., Tokyo, Japan). Column chromatography was performed using silica gel (Kanto, 40–50 μm, Tokyo, Japan), Cosmosil 75C18-OPN (Nacalai Tesque Inc., Kyoto, Japan) and Sephadex LH-20 (Dowex® 50WX2-100, Sigma-Aldrich, Tokyo, Japan). Analytical TLC was performed on pre-coated silica gel 60F_254_ and RP-18 F_254_ plates (0.25 or 0.50 mm thickness, Merck KGaA, Darmstadt, Germany). Cosmosil 5C18-AR-II (Nacalai Tesque Inc., Kyoto, Japan) was used for analytical and semi-preparative HPLC (250 × 4.6 mm for analytical HPLC, and 250 × 10.0 mm for semi-preparative HPLC).

### Plant material

The aerial parts of *H. pilulifera* were collected in the Quang Tri province (17°02'15.2”N 107°03'55.9”E), Vietnam, in August 2014 and were identified by Dr Nguyen The Cuong, Institute of Ecology and Biological Resources, VAST, Vietnam. A voucher specimen (VL01) was deposited at the Faculty of Pharmacy, Hue University of Medicine and Pharmacy, Vietnam.

### Extraction and isolation of constituents

The aerial parts of *H. pilulifera* (1.4 kg) were extracted by soaking with hot MeOH (3 × 3 L, 3 h each, 60 °C) to yield 38.0 g of a dark solid extract. This extract was suspended in water and successively partitioned with chloroform (CHCl_3_) and ethyl acetate (EtOAc) to obtain the CHCl_3_ (HC, 13.0 g), the EtOAc (HE, 11.0 g) and the water (HW, 14.0 g) extracts after the removal of solvents *in vacuo*.

The HC extract was chromatographed on a silica gel column eluting with *n*-hexane:acetone gradient system (100:0, 95:5, 90:10, 50:10, 10:10, 0:100 v/v, each 1.0 L) to obtain 6 corresponding fractions, HC1–HC6. Fraction HC3 (1.9 g) was subjected to a silica gel column eluting with *n*-hexane:EtOAc (6:1, v/v) to obtain 5 sub-fractions (HC3A–HC3E). Fraction HC3B (210 mg) was then purified by silica gel column chromatography eluting with CHCl_3_:acetone (15:1, v/v) to give **2** (11.0 mg), **6** (5.5 mg) and **10** (12.0 mg). Fraction HC3C (180 mg) was loaded onto an open silica gel column eluting with *n*-hexane:CHCl_3_:acetone (6:5:1, v/v) to obtain **5** (8.0 mg) and **12** (7.5 mg). Fraction HC3E (2.1 g) was chromatographed over silica gel column using chloroform:EtOAc (5:1, v/v) as eluent to obtain 6 sub-fractions (HC3E1–HC3E6). Fraction HC3E4 (125 mg) was chromatographed on a silica gel column eluting with *n*-hexane:EtOAc:acetone (5:5:1, v/v) to afford **3** (2.6 mg), **7** (4.9 mg) and **13** (9.0 mg). Fraction HC6 (1.5 g) was subjected to silica gel column eluting with *n*-hexane:CHCl_3_:MeOH (1:3:1, v/v) to give 6 smaller fractions (HC6A–HC6F). Fraction HC6E (360 mg) was further purified by using reverse-phase RP-18 silica gel column eluting with MeOH:water (30:1, v/v) to yield **1** (8.1 mg) and **4** (4.5 mg).

Fraction HE was crudely separated into 4 smaller fractions (HE1–HE4) by silica gel column using CHCl_3_:EtOAc:MeOH (15:15:1, v/v) as eluent. Fraction HE3 (1.8 g) was chromatographed on a silica gel column eluting with CHCl_3_:acetone (10:1, v/v) to yield **11** (4.7 mg) and 3 sub-fractions (HE3A–HE3D). Fraction HE3C was then partitioned on a sephadex LH-20 column eluting with MeOH to furnish **8** (20.5 mg). Compound **9** (3.4 mg) was purified from sub-fraction HE3D by preparative TLC using CHCl_3_:MeOH (15:1, v/v) as a mobile phase. Fraction HE4 (1.1 g) was further subjected to reverse-phase RP-18 silica gel column eluting with MeOH:water (15:1, v/v) to afford **14** (4.5 mg) and 3 smaller fractions (HE4A–HE4C). HE4A was purified by preparative reversed phase HPLC using MeOH:water (95:5, flow rate 3 mL/min) as eluent to afford **17** (6.8 mg), **18** (4.5 mg), **19** (11.0 mg) and **20** (6.0 mg). Finally, HE4B was purified by repeated reverse-phase RP-18 silica gel column using acetone:MeOH:water (3:5:1, v/v) as eluent to give **15** (4.6 mg) and **16** (5.2 mg).

### Bioassay

Antimicrobial activity was performed using the dilution method to a published procedure (Eloff 1998) with slight modifications. *Staphylococcus aureus* (NBRC 100910), *Bacillus subtilis* (NBRC 13719), *Mycobacterium smegmatis* (NBRC 13167) were used for this assay. These strains were tested by using microdilution assays, and MIC values were determined (Eloff 1998). Bacterial strains were inoculated on YP agar plates [1% polypeptone (Nihon Pharmaceutical, Tokyo, Japan), 0.2% yeast extract (Difco, Mich., Detroit, MI), 0.1% MgSO_4 _−_ _7H_2_O, and 2% agar (Nacalai Tesque Inc., Kyoto, Japan)] and incubated at 30 °C for 12 h. A stock solution of samples was prepared at 1 mg/mL in DMSO and further diluted to varying concentrations in 96-well plates that contained microbial strains incubated in YP medium for the bacterial strains. The plate was further incubated at 37 °C for 12 h. Ampicillin (Nacalai Tesque Inc., Kyoto, Japan) were used as the reference reagents for bacterial strains (Eloff 1998).

## Results

The chemical investigations led to the isolation and the structural elucidation of 20 known compounds including five triterpenoids, two steroids, two aromatic compounds, three fatty acids, one quinone derivative, one lignan glycoside, one ceramide and five glycolipids.

Compound **1** was obtained as a white amorphous powder. The HREIMS of **1** showed a molecular ion peak at *m/z* 456.3611 [M]^+^. Its molecular formula was thus determined to be C_30_H_48_O_3_ by HREIMS in conjunction with NMR data analysis. The ^1^H-NMR of **1** showed the presence of seven quaternary methyl groups at *δ*_H_ 0.92 (3H, s), 0.98 (3H, s), 1.03 (3H, s), 1.05 (6H, s), 1.27 (3H, s), 1.31 (3H, s) and one olefinic proton at *δ*_H_ 5.53 (1H, br.s, H-12). The ^13^C-NMR spectrum revealed 30 signals. The presence of one carboxylic group [*δ*_C_ 180.3 (C-28)], one tri-substituted double bond [*δ*_C_ 122.7 (C-12) and 144.9 (C-13)], one oxygenated carbon [*δ*_C_ 78.2 (C-3)] were observed. Based on the above MS and NMR data (Table 1), compound **1** was determined to be oleanolic acid, consistent with reported data (Mahato & Kundu 1994).

**Table 1.** ^1^H (500 MHz) and ^13^C (125 MHz) NMR data of compounds **1**, **4**, and **5** [δ (ppm), *J* (Hz)].

Position	1^a^		4^a^		5^b^	
	*δ_*C*_*	*δ_*H*_*	*δ_*C*_*	*δ_*H*_*	*δ_*C*_*	*δ_*H*_*
1	39.0		39.2	0.96^c^, 1.53^c^	39.1^c^	1.66^c^, 1.77^c^
2	28.2		28.9	1.89^c^, 2.05^c^	27.1	1.28^c^, 1.58^c^
3	78.2	3.48 dd (9.5, 6.0)	80.7	3.64 dd (11.5, 3.5)	74.1	3.64 dd (11.5, 4.5)
4	39.5	−	43.6	–	43.3^d^	–
5	55.9		56.9	1.00^c^	48.7^c^	1.20^c^
6	18.9		19.7	1.38^c^, 1.71^c^	19.3	1.47^c^
7	33.4		34.4	1.38^c^, 1.62^c^	33.7	1.32^c^, 1.68^c^
8	39.9	−	40.8	–	41.0	–
9	48.2		48.3	1.84^c^	48.5^c^	1.77^c^
10	37.5	−	37.6	–	37.9	–
11	23.8		24.7	1.96^c^, 2.08^c^	24.7	2.01^c^, 1.21^c^
12	122.7	5.53 br.s	128.4	5.62 brs	129.4	5.30 m
13	144.9	−	140.4	–	140.1	–
14	42.3	−	42.5	–	42.7	–
15	28.4		29.8	1.30^c^, 2.33m	29.6	1.01^c^, 1.84^c^
16	23.9		26.9	2.07^c^, 3.15 m	26.7	1.58^c^, 2.58 m
17	46.8	−	48.8	–	49.0^c^	–
18	42.1	3.33 dd (13.0, 2.5)	55.1	3.07 s	55.1	2.53 s
19	46.6		73.2	–	73.6	–
20	31.1	−	42.9	1.52^c^	43.1	1.37^c^
21	34.3		27.4	1.36^c^, 1.55^c^	27.4	1.37^c^, 1.77^c^
22	33.3		39.0	2.08^c^, 2.19^c^	39.5	1.03^c^, 1.62^c^
23	28.9	1.27 s	**24.1**	1.56 s	**67.5**	3.57 d (12.0) 3.31^c^
24	16.7	1.05 s	**65.1**	4.51 d (10.5); 3.68 d (10.5)	**12.7**	0.73 s
25	15.6	0.92 s	16.5^d^	0.90 s	16.2^d^	1.00 s
26	17.5	1.03 s	17.5^d^	1.09 s	17.6^d^	0.83 s
27	26.3	1.31 s	25.1	1.74 s	24.9	1.36 s
28	180.3	−	181.2	–	182.9	–
29	33.4	0.98 s	27.6	1.47 s	27.3	1.21 s
30	23.9	1.05 s	17.3^d^	1.14 d (6.5)	16.6^d^	0.95 d (6.0)

a Measured in pyridine-*d*_5_.

b Measured in CD_3_OD.

c Overlapped signals.

d Reassigned using 2D-NMR.

Compound **4** was obtained as a white amorphous powder. The ^1^H-NMR spectrum showed the typical signals of one olefinic proton (*δ*_H_ 5.62), two protons of oxymethylene group [*δ*_H_ 3.68 (d, *J* = 10.5 Hz), 4.51 (d, *J* = 10.5 Hz)], one proton of carbinol group [*δ*_H_ 3.64 (dd, *J* = 11.5, 3.5 Hz)], and seven methyl groups [*δ*_H_ 0.90 (s), 1.09 (s), 1.14 (d, *J* = 6.5 Hz), 1.47 (s), 1.56 (s), and 1.74 (s) (each, 3H)]. The coupling constant value of H-3 (11.5 and 3.5 Hz) is typical for its α-axial orientation in chair conformation of A ring. The ^13^C-NMR spectrum indicated 30 signals. Some characteristic signals were found, including one carbonyl carbon (*δ*_C_ 181.2), two olefinic carbons (*δ*_C_ 128.4 and 140.4), one oxygenated methine carbon (*δ*_C_ 80.7), one oxygenated quaternary carbon (*δ*_C_ 73.2) and one oxygenated methylene carbon (*δ*_C_ 65.1). The complete assignment of all protons and carbons of **4** was conducted by the analysis of HMQC and HMBC spectra (Table 1). The HMBC correlations from methyl protons (*δ*_H_ 1.56) and oxymethylene protons (*δ*_H_ 3.68 and 4.51) to C-3 (*δ*_C_ 80.7)/C-4 (*δ*_C_ 43.6) confirmed that the position of oxygenated methine carbon (C-3) in the A ring. Similarly, the HMBC correlations from H-18 (*δ*_H_ 3.07) and C-12 (*δ*_C_ 128.4)/C-13 (*δ*_C_ 140.4)/C-28 (*δ*_C_ 181.2) confirmed the position of carboxyl group at C-17 as well as the tri-substituted double bond at C-12/C-13. In addition, the HMBC correlation between H_3_-29 (*δ*_H_ 1.47) and C-18 (*δ*_C_ 55.1)/C-19 (*δ*_C_ 73.2)/C-20 (*δ*_C_ 42.9) led us to locate the hydroxyl group at C-19. Next, the remaining hydroxyl group was linked to C-24 by comparison of the chemical shifts of methyl carbon (*δ*_C_ 24.1) and oxymethylene carbon (*δ*_C_ 65.1) with those of the two stereoisomers [24-OH: *δ*_C_ 23.5 (C-23)/64.5 (C-24); 23-OH: *δ*_C_ 67.9 (C-23)/12.8 (C-24)] (Zhang & Yang 1994). Consequently, compound **4** was concluded to be rotungenic acid (Nakatani et al. 1989).

Compound **5** was obtained as a white amorphous powder. The ^1^H and ^13^C NMR chemical shifts of **5** were almost the same as those of **4** (Table 1), suggesting that these two compounds possessed the analogous structures. The only difference is configuration of chiral centre at C-4. Similar to **4**, the hydroxyl group was located at C-23 due to the chemical shifts of methyl carbon (*δ*_C_ 12.7) and oxymethylene carbon (*δ*_C_ 67.5) at C-4 (*δ*_C_ 43.3) were similar to those of 23-OH form [*δ*_C_ 67.9 (C-23)/12.8 (C-24)], but quite different from those of 24-OH form [*δ*_C_ 23.5 (C-23)/64.5 (C-24)] (Zhang & Yang 1994). Thus, compound **5** was identified to be rotundic acid (He et al. 2012).

**Figure 1.**
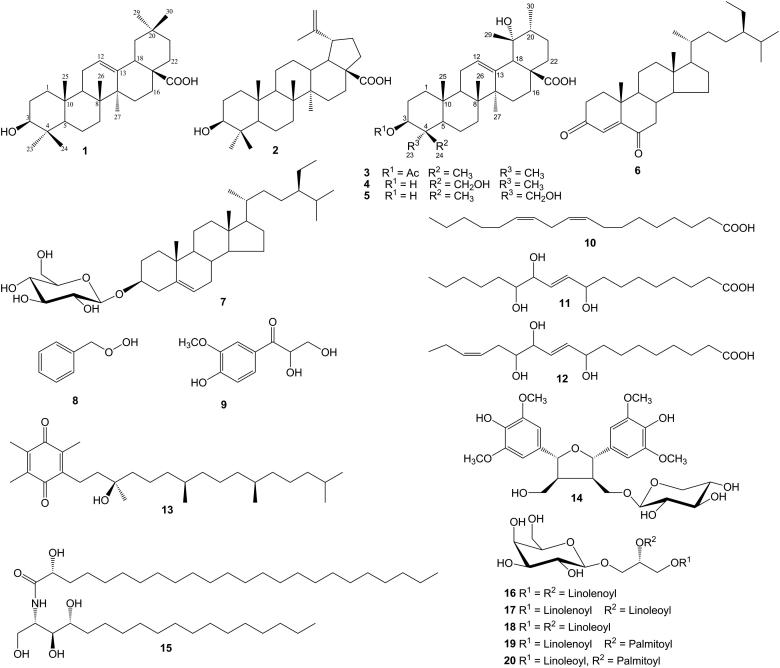
Chemical structures of isolated compounds (**1**–**20**): oleanolic acid (**1**), betulinic acid (**2**), pomolic acid 3β-acetate (**3**), rotungenic acid (**4**), rotundic acid (**5**), stigmast-4-ene-3,6-dione (**6**), daucosterol (**7**), benzyl hydroperoxide (**8**), 2,3-dihydroxy-1-(4-hydroxy-3-methoxyphenyl)-propan-1-one (**9**), octadeca-9Z,12Z-dienoic acid (**10**), 9,12,13-trihydroxyoctadeca-10E-enoic acid (**11**), 9,12,13-trihydroxyoctadeca-10E,15Z-dienoic acid (**12**), α-tocopherolquinone (**13**), (7S*,8R*,7′R*,8′S*)-icariol A2-9-*O*-β-xylopyranoside (**14**), (2S,3S,4R,2′R)-2-(2′-hydroxytetracosanoylamino)octadecane-1,3,4-triol (**15**), (2S)-1-*O*-linolenoyl-2-*O*-linolenoyl-3-*O*-β-d-galactopyranosyl-sn-glycerol (**16**), (2S)-1-*O*-linolenoyl-2-*O*-linoleoyl-3-*O*-β-d-galactopyranosyl-sn-glycerol (**17**), (2S)-1-*O*-linoleoyl-2-*O*-linoleoyl-3-*O*-β-d-galactopyranosyl-sn-glycerol (**18**), (2S)-1-*O*-linolenoyl-2-*O*-palmitoyl-3-*O*-β-d-galactopyranosyl-sn-glycerol (**19**), and (2S)-1-*O*-linoleoyl-2-*O*-palmitoyl-3-*O*-β-d-galactopyranosyl-sn-glycerol (**20**).

The remaining compounds were identified as betulinic acid (**2**) (Hess & Monache 1999), pomolic acid 3β-acetate (**3**) (Neto et al. 2000), stigmast-4-ene-3,6-dione (**6**) (Seca et al. 2000), daucosterol (**7**) (Yang et al. 2013), benzyl hydroperoxide (**8**) (Kyasa et al. 2013), 2,3-dihydroxy-1-(4-hydroxy-3-methoxyphenyl)-propan-1-one (**9**) (Baderschneider & Winterhalter 2001), octadeca-9*Z*,12*Z*-dienoic acid (**10**) (Butovich & Lukyanovaon 2008), 9,12,13-trihydroxyoctadeca-10*E*-enoic acid (**11**) (Oueslati et al. 2006), 9,12,13-trihydroxyoctadeca-10*E*,15*Z*-dienoic acid (**12**) (Oueslati et al. 2006), α-tocopherolquinone (**13**) (Sung et al. 1999), (7S*,8R*,7′R*,8′S*)-icariol A_2_-9-*O*-β-xylopyranoside (**14**) (Chung et al. 2011), (2*S*,3*S*,4*R*,2′*R*)-2-(2′-hydroxytetracosanoylamino)octadecane-1,3,4-triol (**15**) (Gao et al. 2001), **(**2*S*)-1-*O*-linolenoyl-2-*O*-linolenoyl-3-*O*-β-d-galactopyranosyl-*sn*-glycerol (**16**) (Kiem et al. 2012), **(**2*S*)-1-*O*-linolenoyl-2-*O*-linoleoyl-3-*O*-β-d-galactopyranosyl-*sn*-glycerol (**17**) (Ibrahim et al. 2011), **(**2*S*)-1-*O*-linoleoyl-2-*O*-linoleoyl-3-*O*-β-d-galactopyranosyl-*sn*-glycerol (**18**) (Janwitayanuchit et al. 2003), **(**2*S*)-1-*O*-linolenoyl-2-*O*-palmitoyl-3-*O*-β-d-galactopyranosyl-*sn*-glycerol (**19**) (Murakami et al. 1991) and **(**2*S*)-1-*O*-linoleoyl-2-*O*-palmitoyl-3-*O*-β-d-galactopyranosyl-*sn*-glycerol (**20**) (Murakami et al. 1991) by comparing their spectroscopic data with those reported in the literature. The chemical structures of all isolated compounds are shown in Figure 1.

The antibacterial assay of isolated compounds was evaluated against three Gram (+) bacterial strains, including *Staphylococcus aureus*, *Bacillus subtilis* and *Mycobacterium smegmatis*. As shown in Table 2, compound **4** demonstrated potent inhibition with MIC values ranging from 1.25 to 2.5 μg/mL. The inhibitory effect of **4** against the tested bacterial strains was comparable with that of the positive control, ampicillin. Compounds **1** and **5** exhibited significant antibacterial activity against *M. smegmatis*, *B. subtilis* with MIC values of 2.5, and 5 μg/mL, respectively. The other compounds (**2**, **6**, and **15**) showed moderate inhibitory effect.

**Table 2.** Antibacterial activities of compounds **1, 2, 4–6**, and **15**.

Compounds	MIC (μg/mL)		
	*S. aureus*	*B. subtilis*	*M. smegmatis*
**1**	10	>20	2.5
**2**	20	20	>20
**4**	2.5	2.5	1.25
**5**	10	**5**	>20
**6**	>20	20	>20
**15**	20	> 20	>20
Ampicillin	5	10	10

## Discussion

To the best of our knowledge, compounds **3–6**, **8–9** and **11–20** were firstly isolated from the genus *Hedyotis.* From our understanding, the antibacterial activity of compounds **4**, **6**, and **15** was reported for the first time in this study.

The structure–activity relationship of **4** and **5** may be due to the difference in absolute configuration of C-4 under the experimental conditions. The 4*S* configuration (in **4**) was considered to be more active than 4*R* configuration (in **5**). Compounds **1**, **4**, and **5** represent pentacyclic triterpenoid skeleton which should be responsible for the bioactivity of the methanol extract of this species.

Previous studies discovered the antibacterial activity of **1** against *Enterococcus faecium*, *Streoptococcus pneumoniae, Staphylococcus aureus* (Horiuchi et al. 2007; Jo et al. 2014), *Listeria monocytogenes* (Hee 2015), *Streptococcus downei* (Park & Kim 2011), *Streptococcus mutans* and *S. sobrinus* (Kim et al. 2010) and *Propionibacterium acnes* (Jo et al. 2014), whereas compound **2** exhibited mild inhibitory effect against *B. subtilis* (Chandramu et al. 2003). Moreover, compound **5** showed broad antimicrobial activity against bacteria, yeast and filamentous fungi (Haraguchi et al. 1999).

## Conclusions

Based on the obtained results, oleanolic acid (**1**), rotungenic acid (**4**), and rotundic acid (**5**) from the aerial parts of *H. pilulifera* were considered to be useful for developing new antimicrobial therapeutic agents for human.

## Supplementary Material

Ain_Raal_et_al_supplemental_content.zip
