# Supplementary material for: Antibacterial activities of chemical constituents from the aerial parts of Hedyotis pilulifera
Source: Pharm Biol. 2017 Jan 19;55(1):787–91. doi: 10.1080/13880209.2017.1279673 (PMC6130504; doi:10.1080/13880209.2017.1279673)
Supplement: Ain_Raal_et_al_supplemental_content.zip [file IPHB_A_1279673_SM4852.zip › Ain Raal et al supplemental content.pdf]

List of Supporting Information

Figure S1. ^{1}H-NMR (500 MHz) spectrum of compound **1** in pyridine-d_5

Figure S2. ^{13}C-NMR (125 MHz) spectrum of compound **1** in pyridine-d_5

Figure S3. ^{1}H-NMR (500 MHz) spectrum of compound **2** in pyridine-d_5

Figure S4. ^{13}C-NMR (125 MHz) spectrum of compound **2** in pyridine-d_5

Figure S5. ^{1}H-NMR (500 MHz) spectrum of compound **4** in pyridine-d_5

Figure S6. ^{13}C-NMR (125 MHz) spectrum of compound **4** in pyridine-d_5

Figure S7. HMQC spectrum of compound **4**

Figure S8. HMBC spectrum of compound **4**

Figure S9. ^{1}H-NMR (500 MHz) spectrum of compound **5** in CD_3OD

Figure S10. ^{13}C-NMR (125 MHz) spectrum of compound **5** in CD_3OD

Figure S11. HMQC spectrum of compound **5**

Figure S12. HMBC spectrum of compound **5**

Figure S13. ^{1}H-NMR (500 MHz) spectrum of compound **6** in $CDCl_3$

Figure S14. ^{13}C-NMR (125 MHz) spectrum of compound **6** in $CDCl_3$

Figure S15. HMQC spectrum of compound **6**

Figure S16. HMBC spectrum of compound **6**

Figure S17. ^{1}H-NMR (500 MHz) spectrum of compound **15** in pyridine-d_5

Figure S18. ^{13}C-NMR (125 MHz) spectrum of compound **15** in pyridine-d_5

Figure S19. HMQC spectrum of compound **15**

Figure S20. HMBC spectrum of compound **15**

Figure S21. COSY spectrum of compound **15**

For Peer Review Only

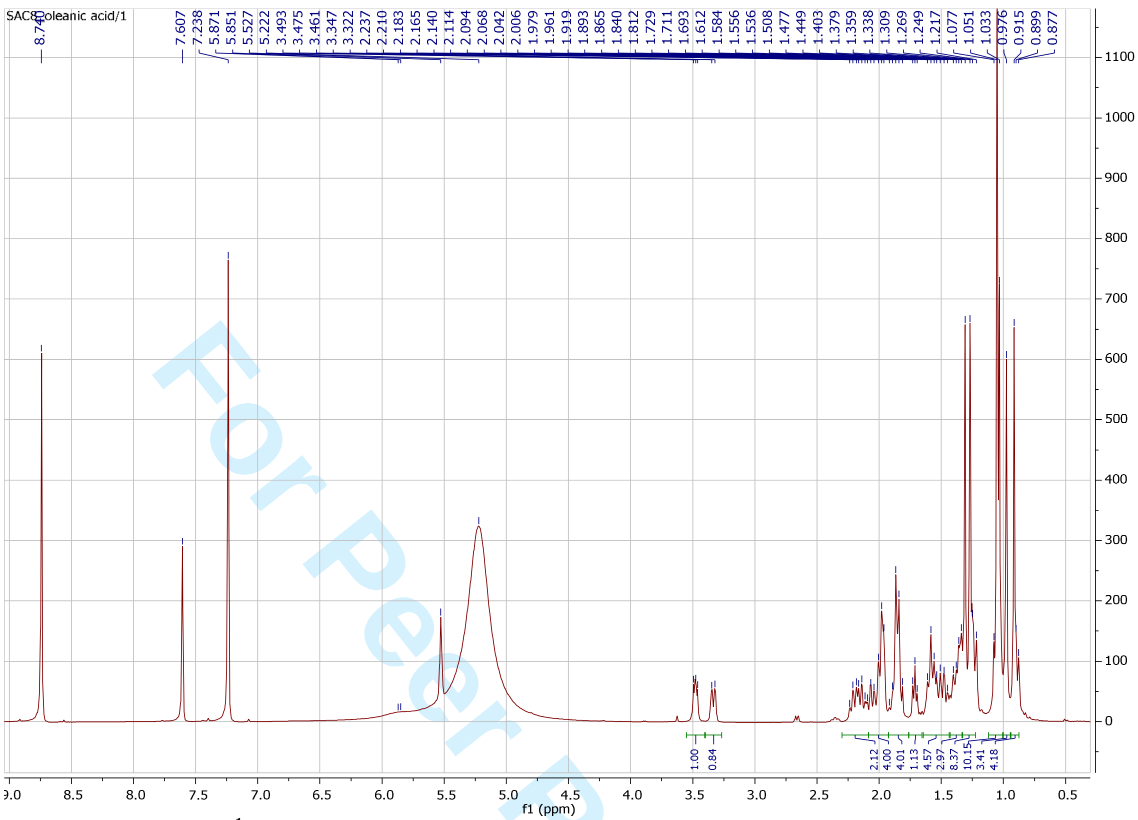

Figure S1.^{1}H-NMR (500 MHz) spectrum of compound **1** in pyridine-d_5

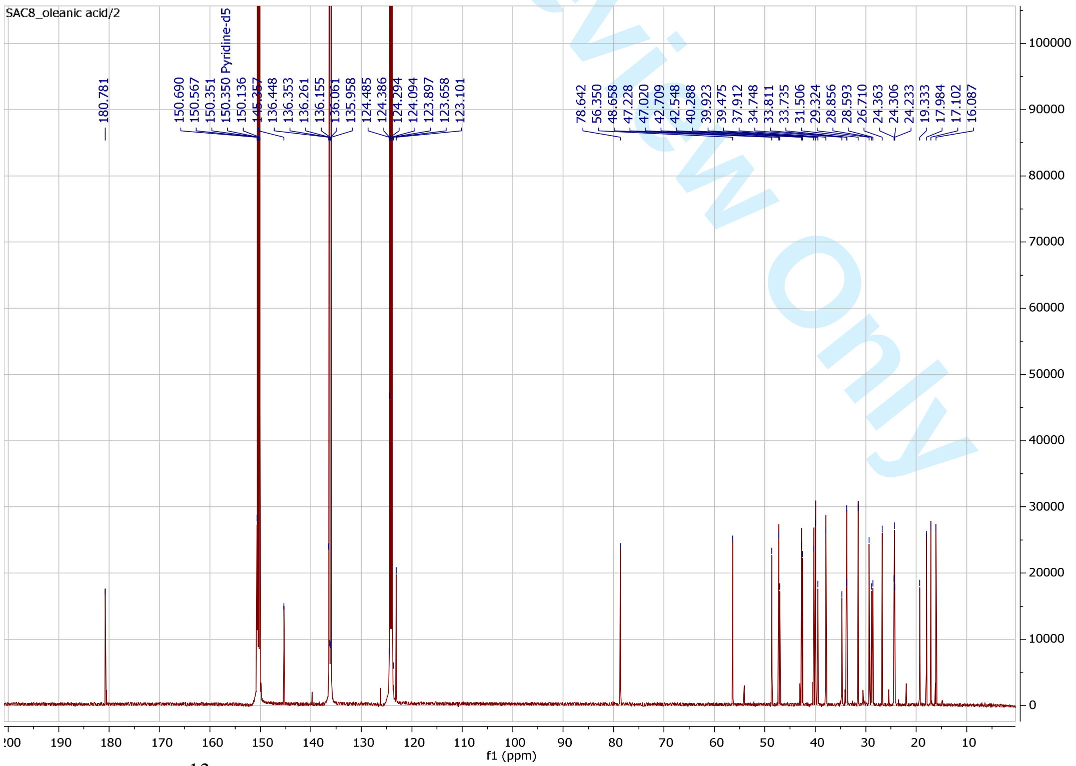

Figure S2.^{13}C-NMR (125 MHz) spectrum of compound **1** in pyridine-d_5

For Peer Review Only

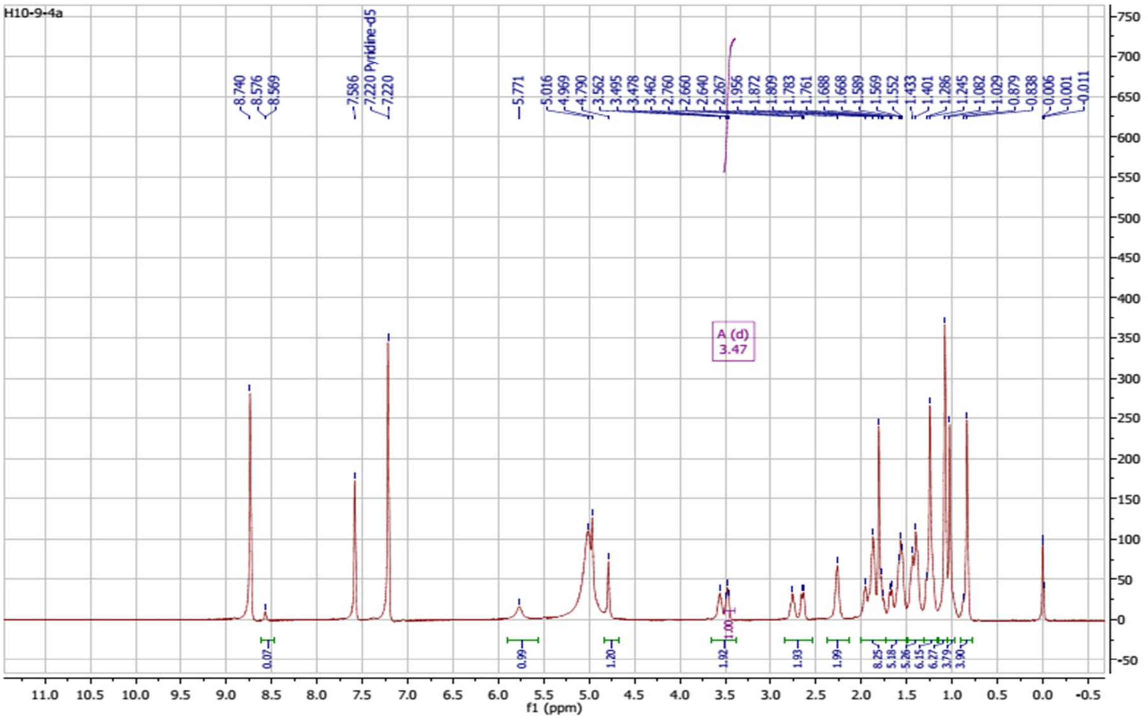

Figure S3. ^{1}H-NMR (500 MHz) spectrum of compound **2** in pyridine-d_5

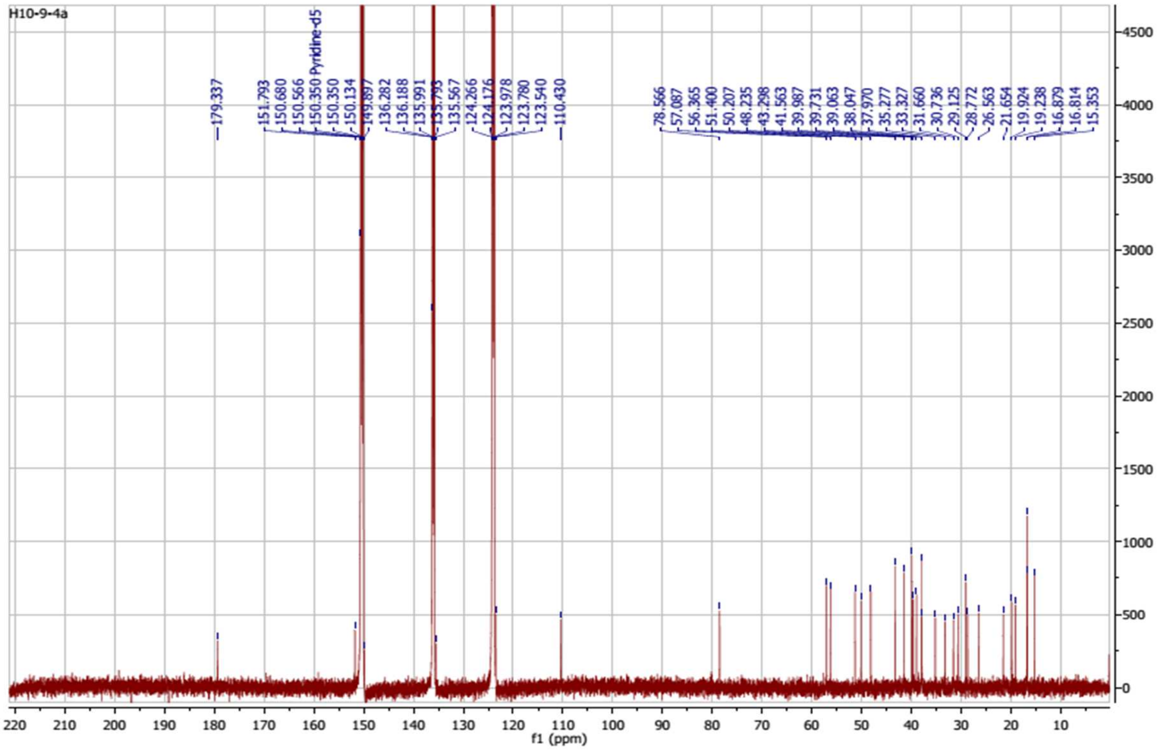

Figure S4. ^{13}C-NMR (125 MHz) spectrum of compound **2** in pyridine-d_5

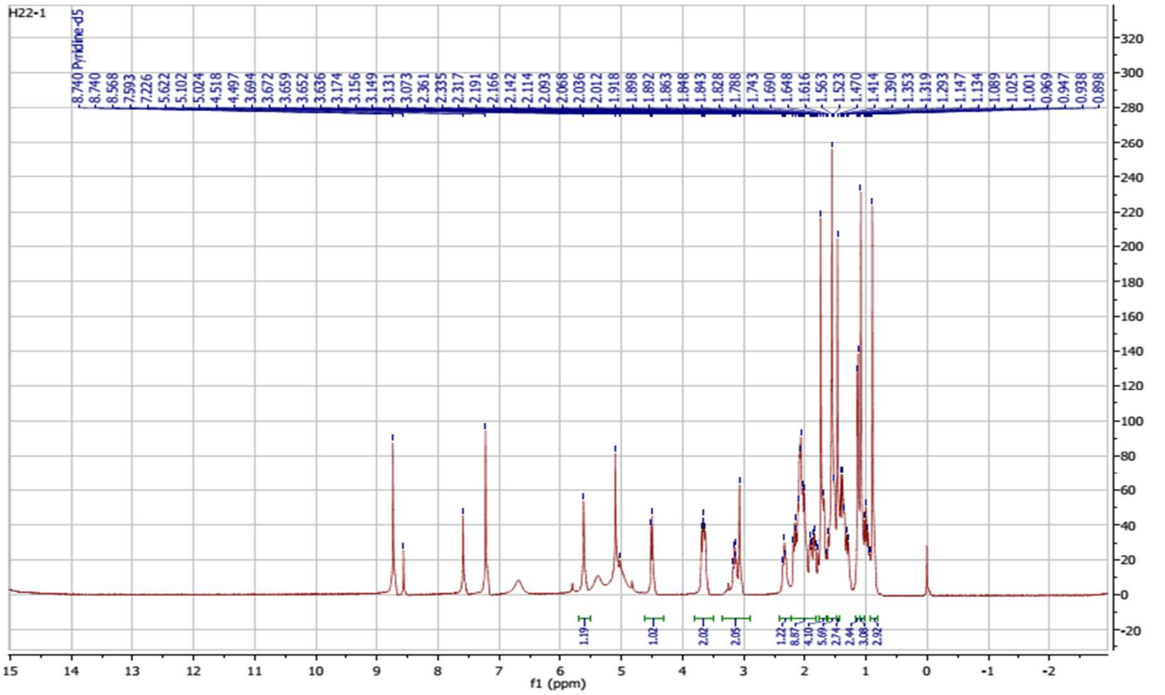

Figure S5. ^{1}H-NMR (500 MHz) spectrum of compound **4** in pyridine-d_5

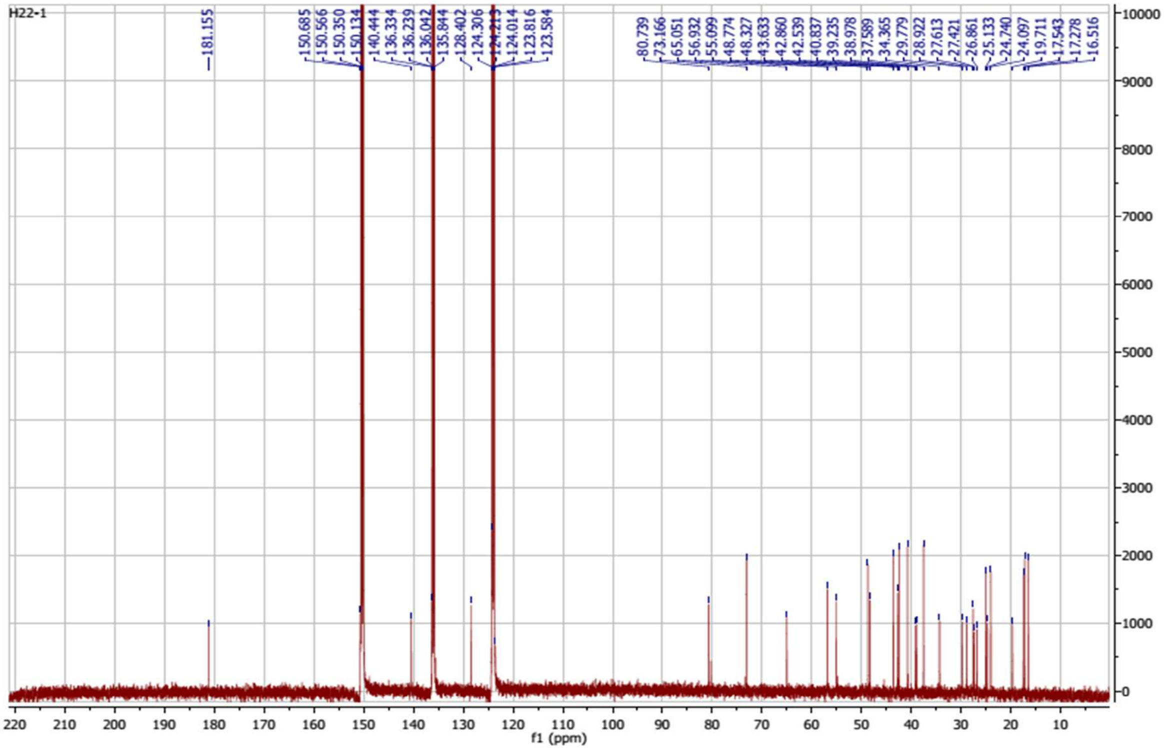

Figure S6. ^{13}C-NMR (125 MHz) spectrum of compound **4** in pyridine-d_5

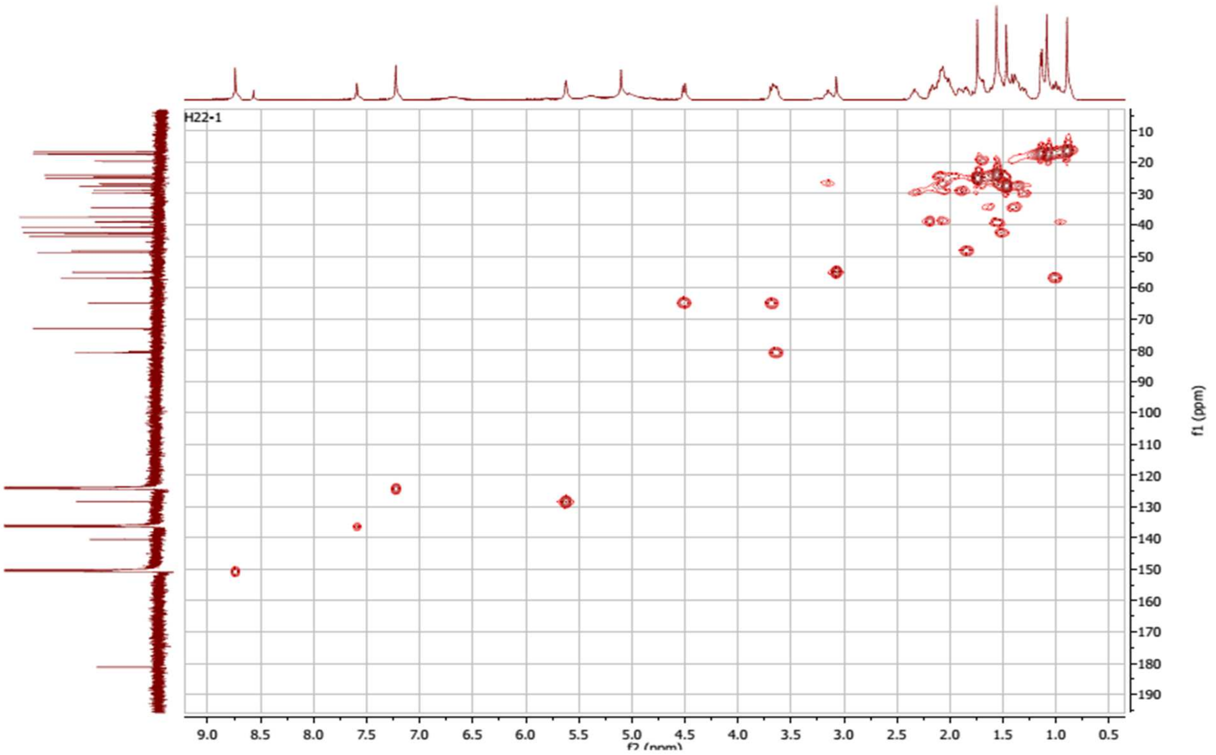

Figure S7.HMQC spectrum of compound **4**

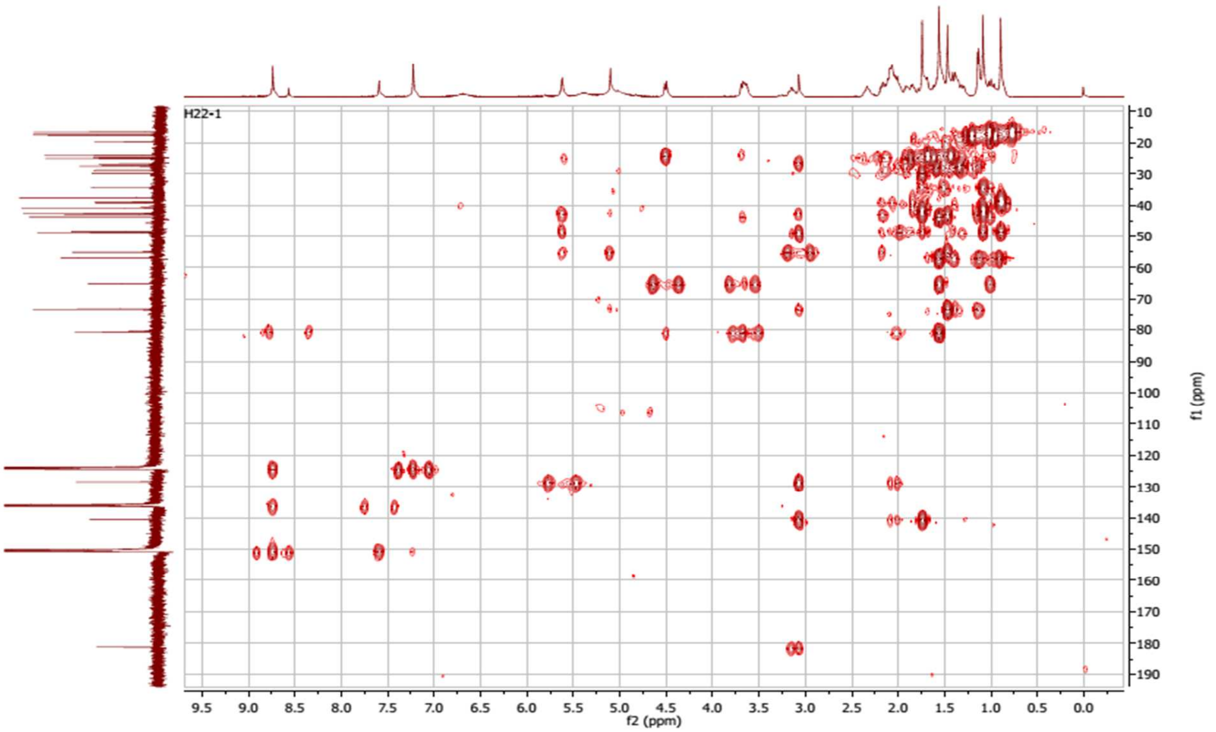

Figure S8.HMBC spectrum of compound **4**

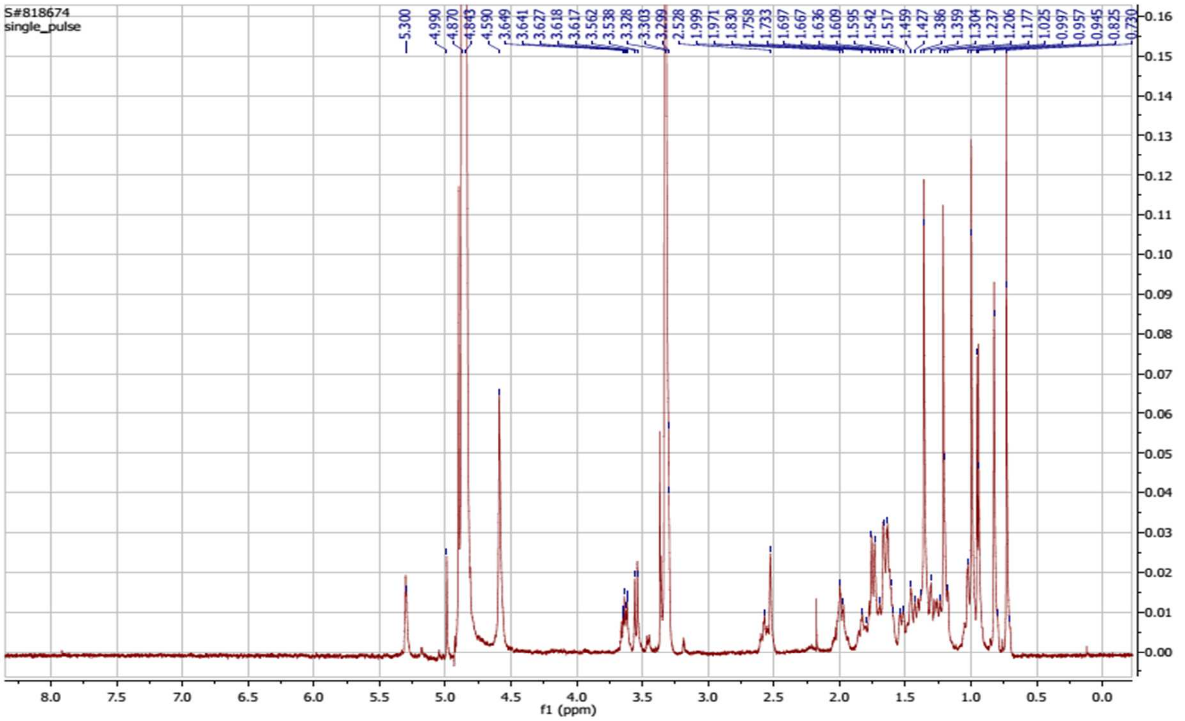

Figure S9. ^{1}H-NMR (500 MHz) spectrum of compound **5** in CD_3OD

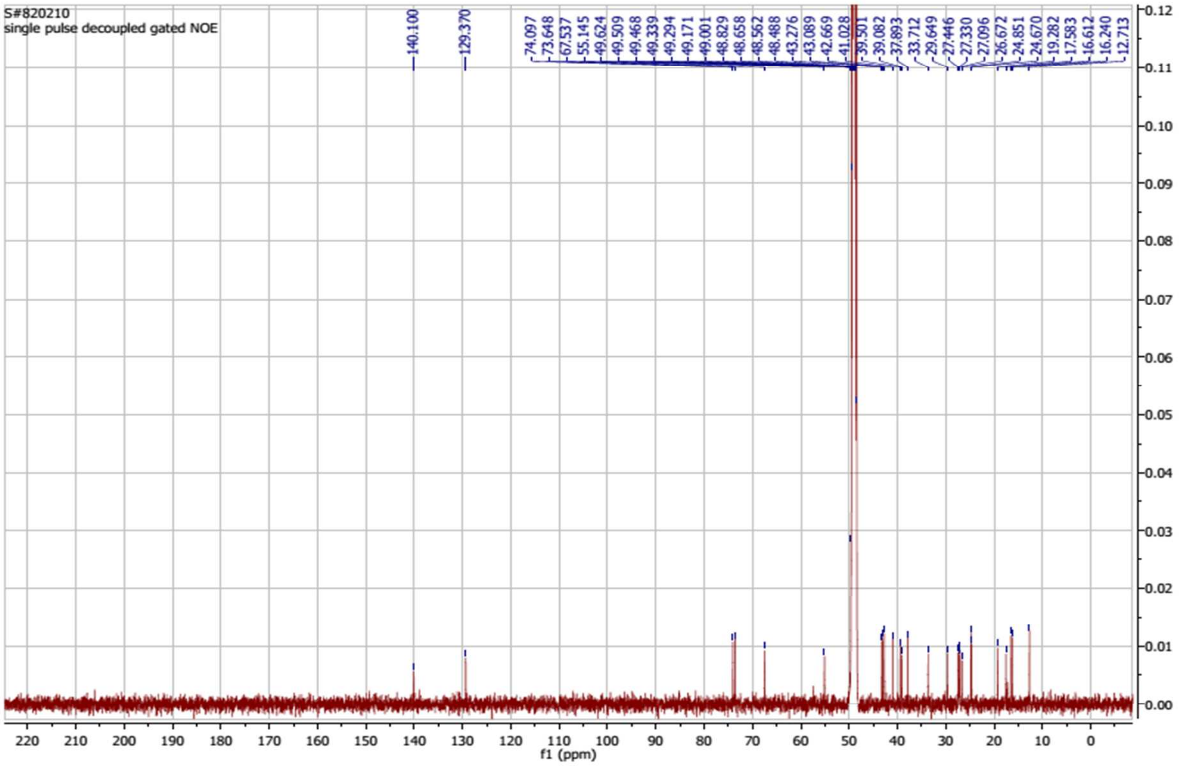

Figure S10. ^{13}C-NMR (125 MHz) spectrum of compound **5** in CD_3OD

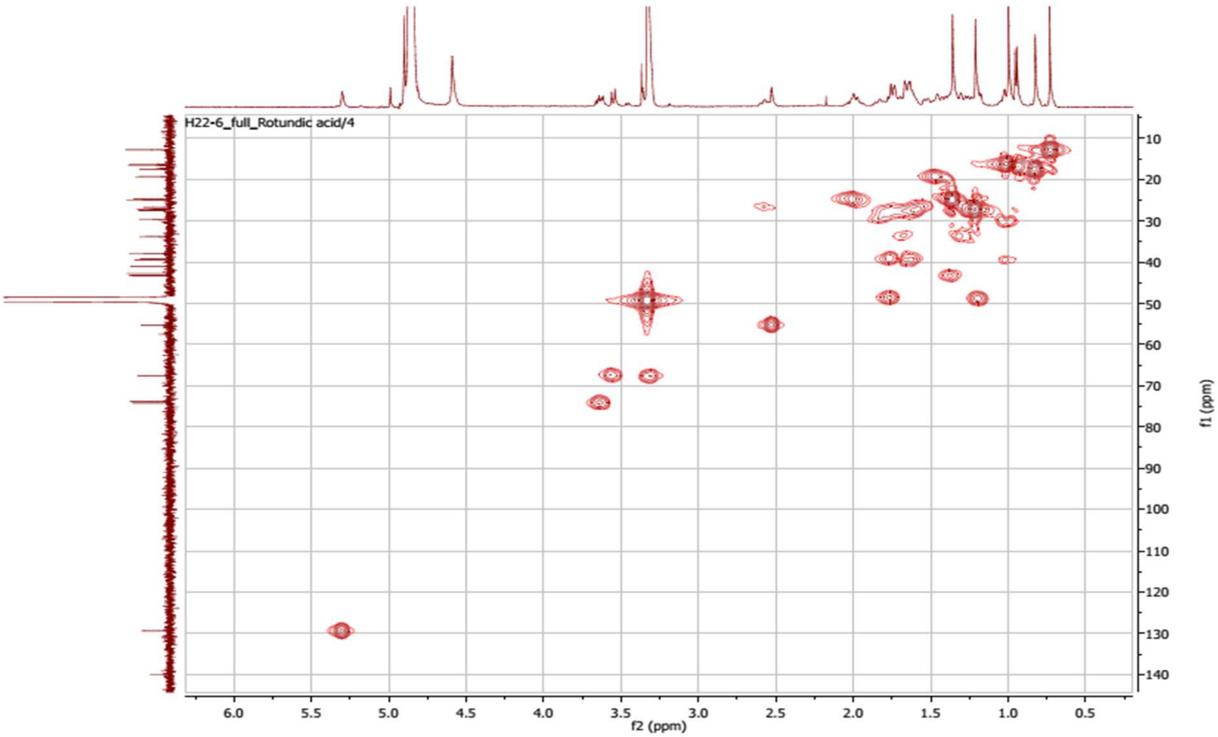

Figure S11.HMQC spectrum of compound **5**

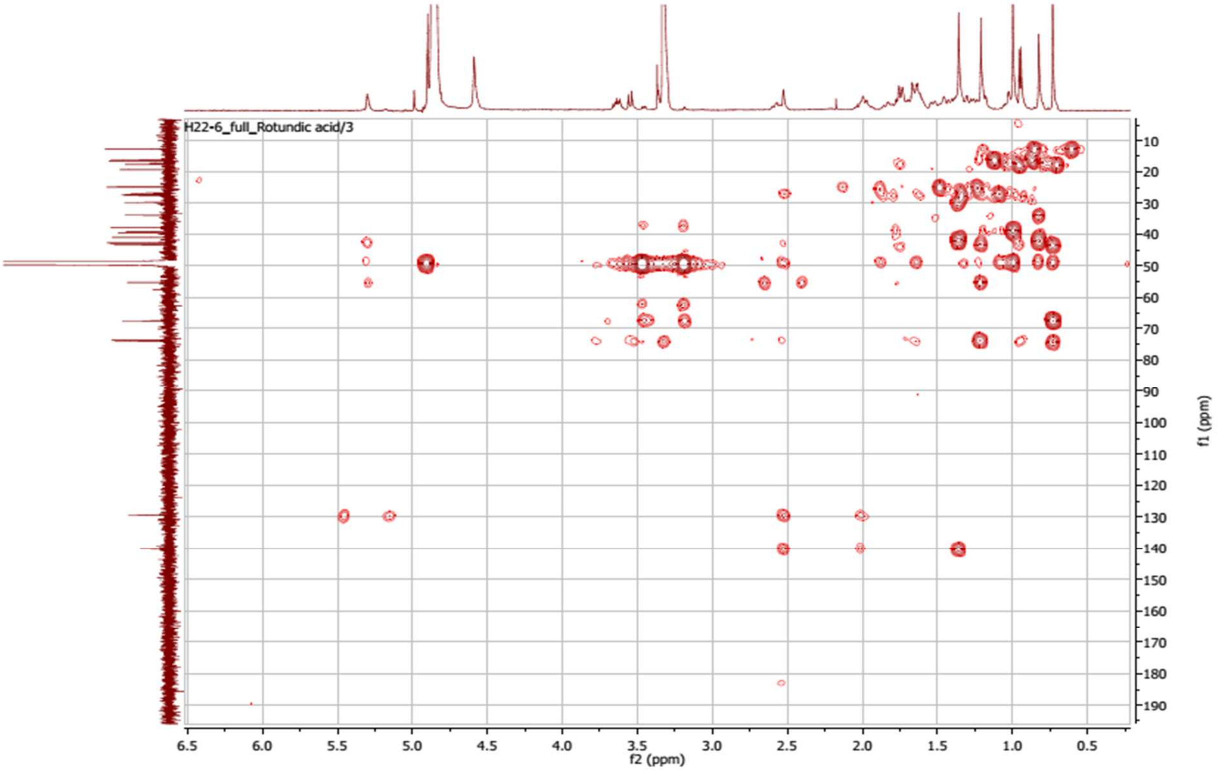

Figure S12.HMBC spectrum of compound **5**

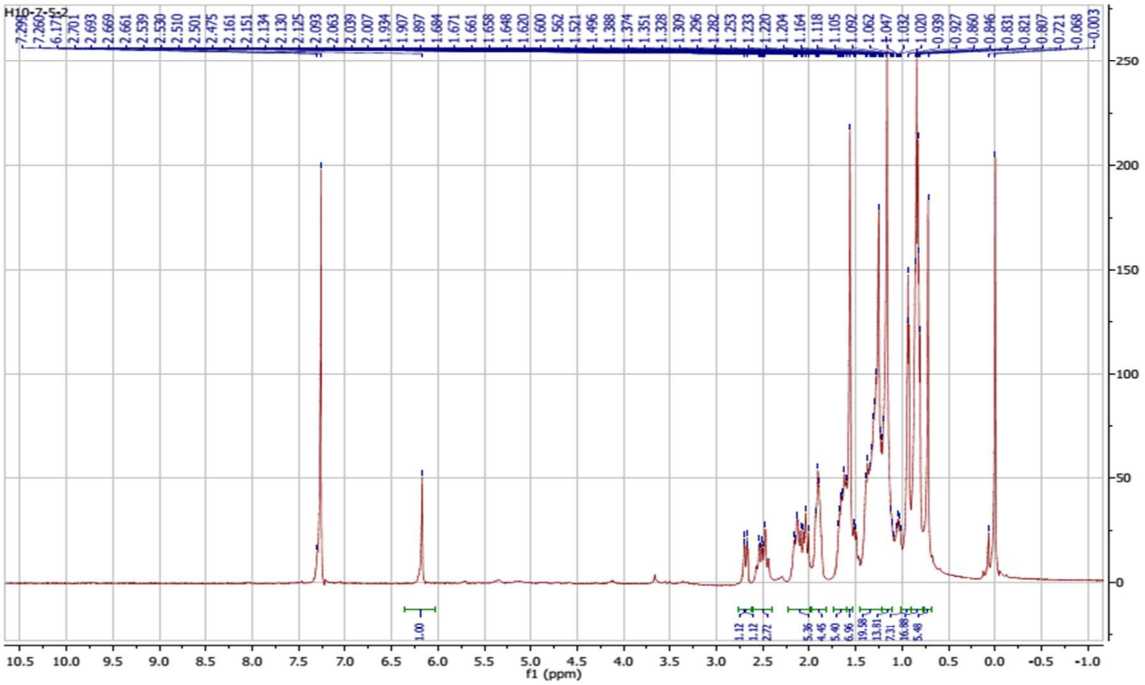

Figure S13.^{1}H-NMR (500 MHz) spectrum of compound **6** in $CDCl_3$

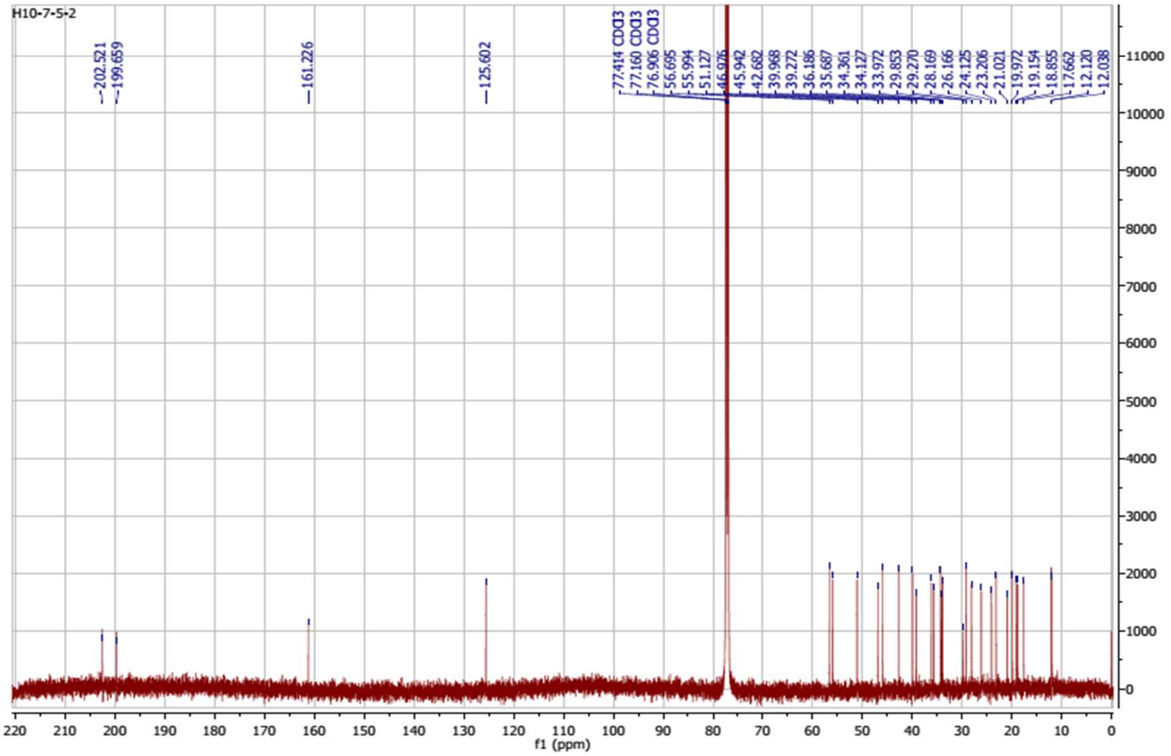

Figure S14.^{13}C-NMR (125 MHz) spectrum of compound **6** in $CDCl_3$

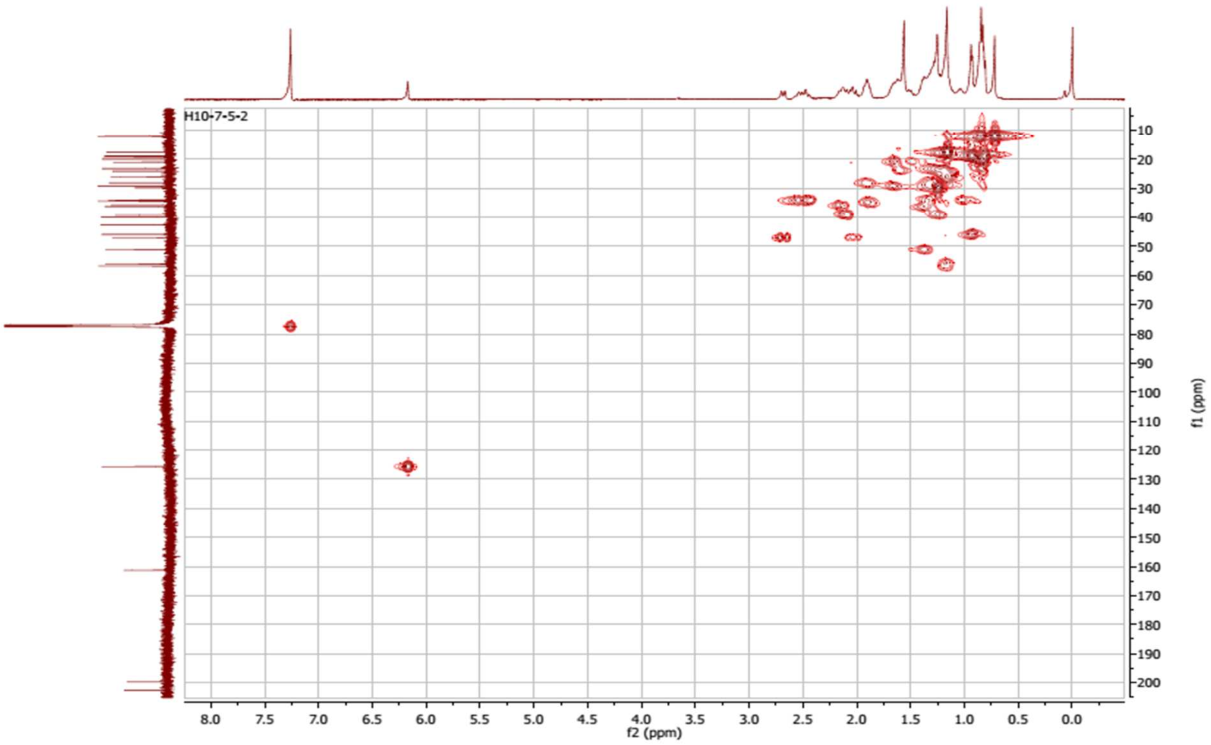

Figure S15.HMQC spectrum of compound **6**

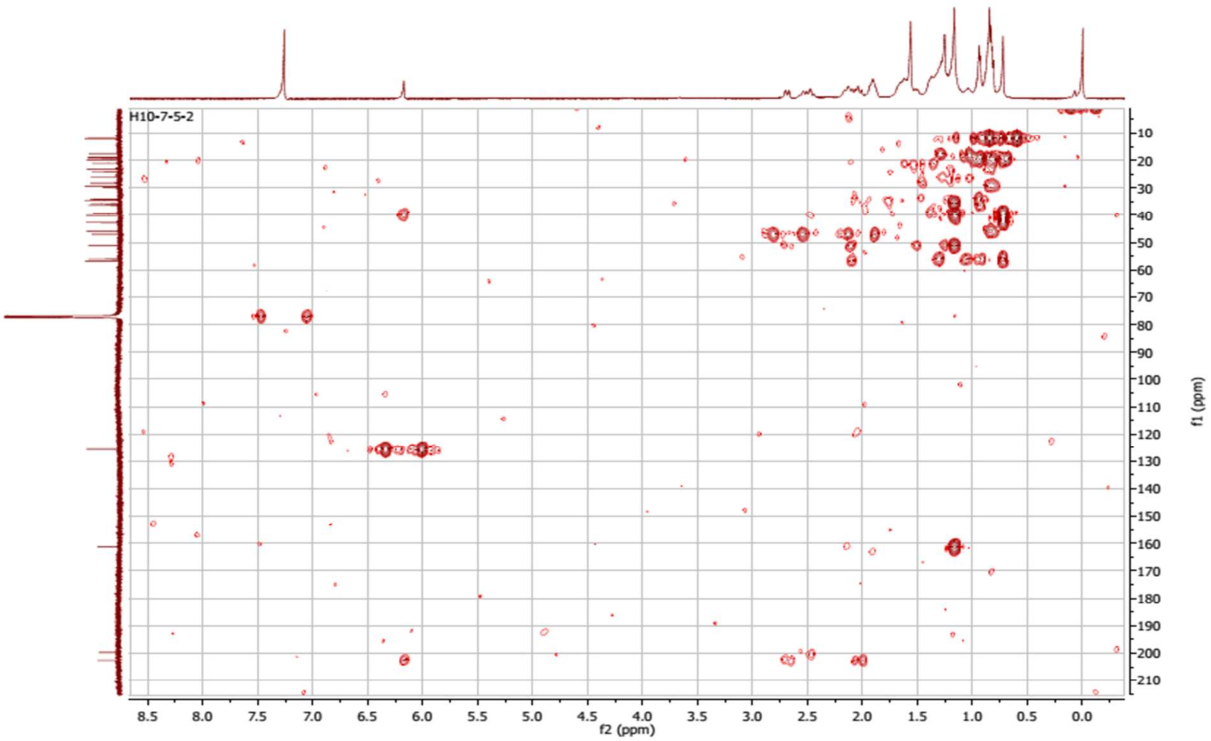

Figure S16.HMBC spectrum of compound **6**

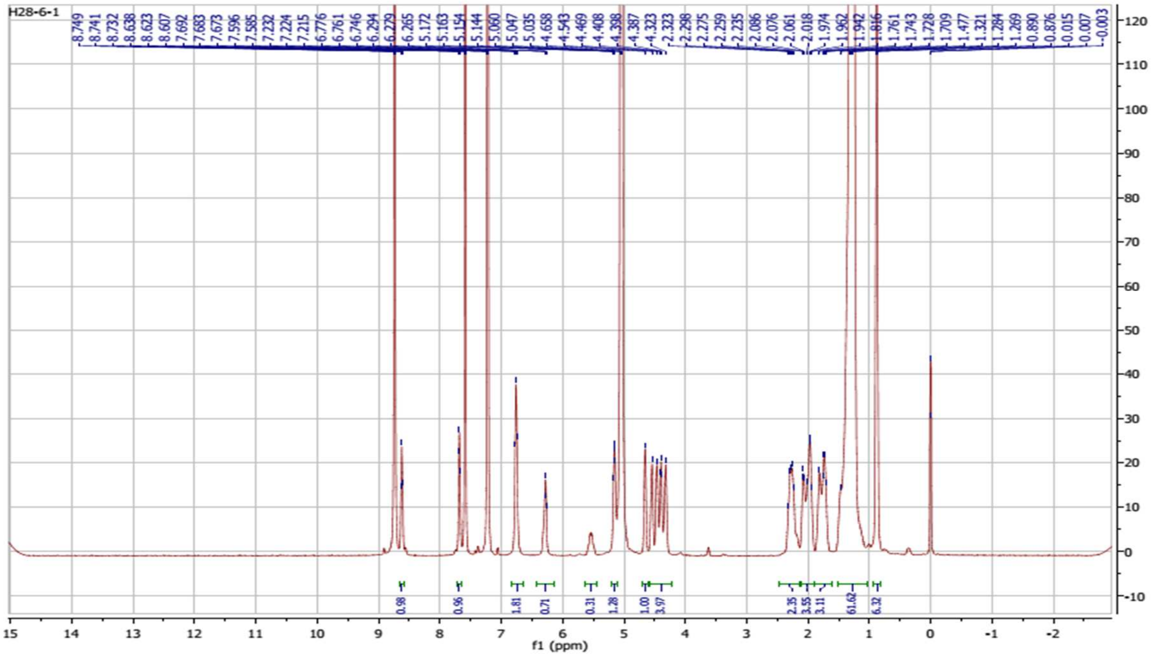

Figure S17. ^{1}H-NMR (500 MHz) spectrum of compound **15** in pyridine-d_5

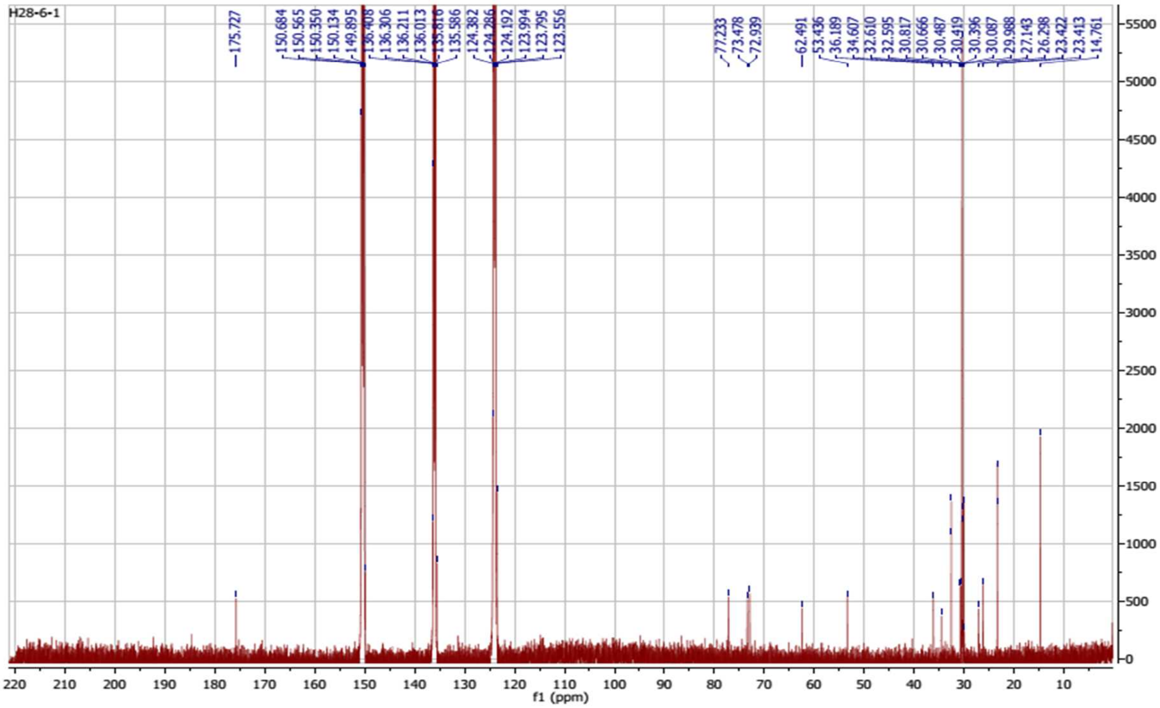

Figure S18. ^{13}C-NMR (125 MHz) spectrum of compound **15** in pyridine-d_5

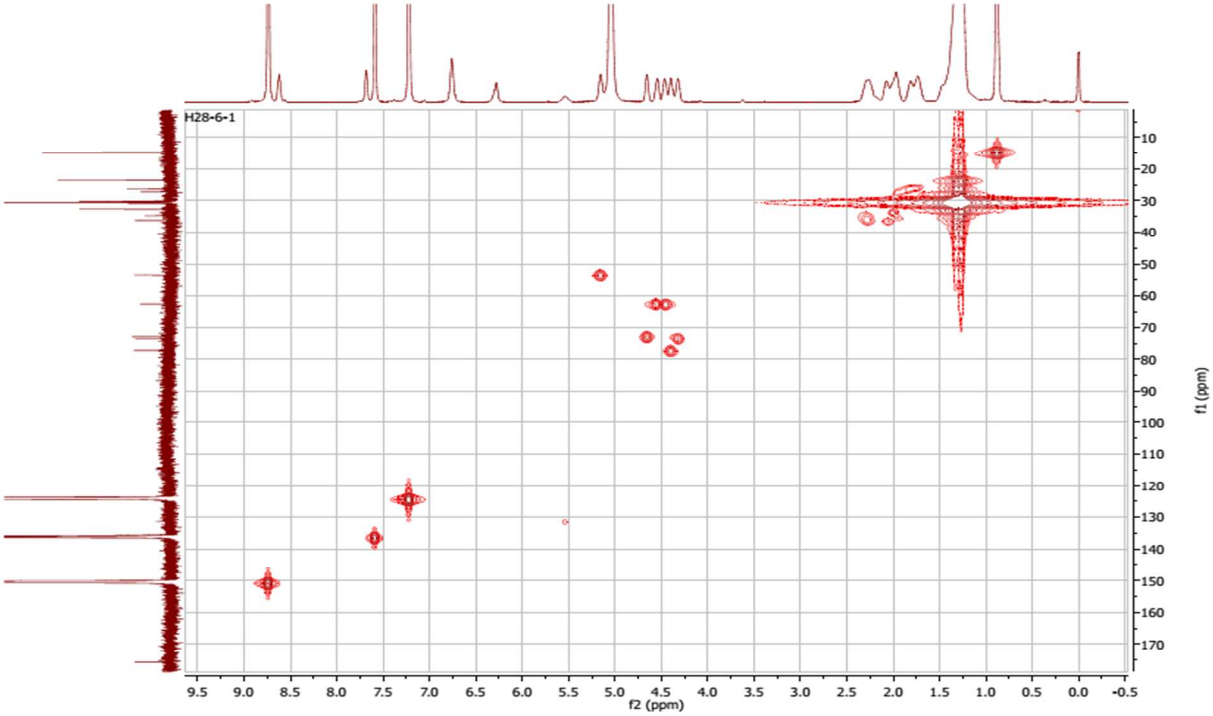

Figure S19.HMQC spectrum of compound **15**

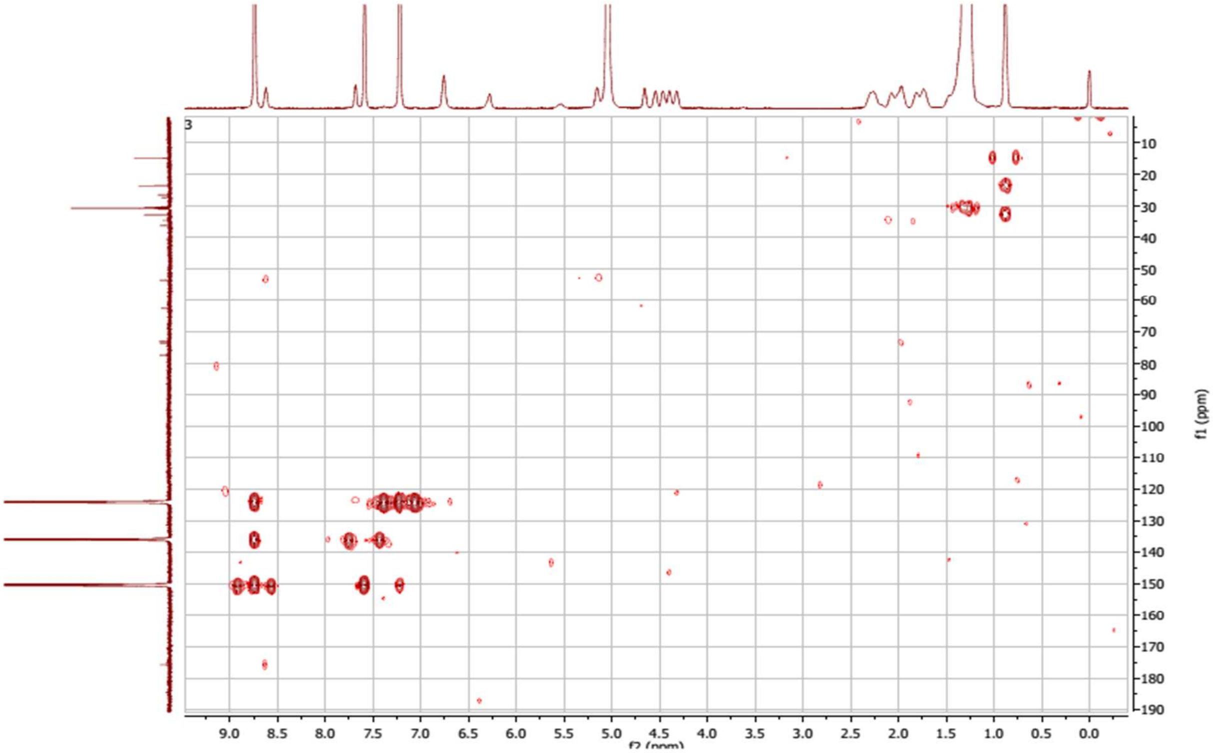

Figure S20.HMBC spectrum of compound **15**

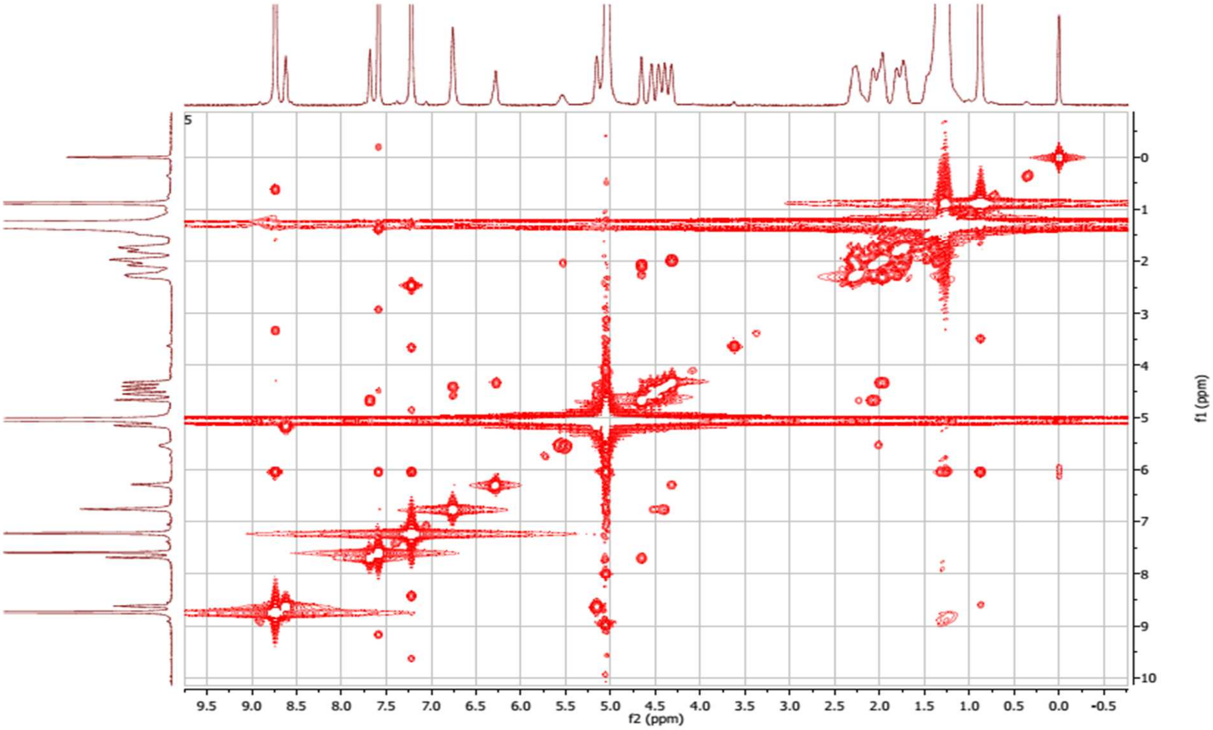

Figure S21.COSY spectrum of compound **15**

Review Only
